# Understanding Risks in the Care of People With Dementia in Acute Hospital Settings: Definitions, Types and Management Strategies—A Scoping Review

**DOI:** 10.1111/jocn.70433

**Published:** 2026-08-08

**Authors:** Ashitha Bhaskaran, Kate Irving, Eileen Courtney, Bert Gordijn, Marie Cantwell

**Affiliations:** ^1^ School of Nursing, Psychotherapy and Community Health Dublin City University Dublin Ireland; ^2^ Wellcome HRB Clinical Research Facility, Trinity College Dublin St James's Hospital Dublin Ireland; ^3^ Dublin City University Dublin Ireland; ^4^ Health Service Executive Dublin Ireland

## Abstract

**Aims:**

This scoping review explores how risk is defined, managed and mitigated in the care of people with dementia in acute hospital settings.

**Methods:**

This scoping review followed Arksesy and O'Malley's five‐stage framework (2005) and was guided by the Joanna Briggs Institute methodology. A literature search was conducted in October 2023 and updated in December 2024 across Web of Science, MEDLINE, CINAHL, Academic Search Complete and PsycINFO databases. The results were reported as per PRISMA‐ScR guidelines.

**Results:**

A total of 3916 articles were screened, 181 full‐text articles assessed and 61 studies included in the final review. The literature on the care of people with dementia in acute hospital settings demonstrates an implicit understanding of risk, focusing mainly on physical risks with less emphasis on psychological, social and spiritual aspects. Identified risk management strategies include targeted screening, dementia‐specific interventions, environmental modifications, non‐pharmacological approaches, person‐centred family‐inclusive care and workforce competency enhancement. Limited studies address positive risk‐taking, and ethical considerations are often overlooked, indicating a need for a more balanced approach to risk in the care of people with dementia in acute settings.

**Conclusion:**

This review highlights critical gaps in the conceptualisation and management of risk in the care of people with dementia in acute settings. A dominant focus on physical safety often overshadows other essential aspects of well‐being, and ethical issues are rarely discussed. The findings underscore the urgent need for person‐centred, ethically informed approaches that balance safety with autonomy and quality of life.

**Patient or Public Contribution:**

No patient or public involvement was included in this review.

**Implications for the Profession:**

This review addresses significant gaps in understanding and managing risks in the care of people with dementia in acute settings. It serves as a vital resource for healthcare professionals to better understand risks in the care of people with dementia in acute settings and consolidate actionable insights that support the delivery of high‐quality, person‐centred care for patients with dementia.

**Trial Registration:**

Open Science Framework: https://doi.org/10.17605/OSF.IO/K67S4

## Contribution of This Paper to the Global Clinical Community

- This scoping review makes a novel contribution to the evidence base on how risks in acute care for people with dementia are defined, described and mitigated. By integrating findings from diverse acute clinical settings, the review addresses a significant gap in understanding the complexity and multifactorial nature of dementia‐related risks in these settings.
- This review provides a novel contribution by systematically synthesising and mapping various risks and risk mitigation strategies in the care of people with dementia in acute settings. It serves as a vital resource for healthcare professionals, hospital administrators and policymakers to better understand the risks associated with caring for people with dementia and consolidate actionable insights that support the delivery of high‐quality, person‐centred care for patients with dementia.
- This work has broader implications for developing and refining policies, guidelines, and training programs to improve the care of people with dementia in acute settings globally.

## 1 Introduction

Dementia is a syndrome characterised by a progressive decline in cognitive function caused by damage or disease in the brain beyond what might be expected from normal ageing (Alzheimer's Association 2021). It encompasses impairments in memory, language, thinking, orientation, comprehension, calculation, learning capacity and judgement (WHO 2021). With an estimated 55 million people living with dementia globally—a number expected to rise to 78 million by 2030 and 139 million by 2050—it is a growing public health concern requiring urgent attention (WHO 2021).

This growing burden is also evident within acute hospital settings, where dementia is highly prevalent among older adults. In Ireland, Timmons et al. (2015) reported a prevalence of approximately 29% among adults aged over 70 years admitted to public acute hospitals, while studies conducted in other settings have reported prevalence rates of up to 42% (Sampson et al. 2009). Hospital admission can trigger distress, confusion and delirium for someone with dementia (NICE 2019). Furthermore, hospital admission frequently leads to worsening behavioural and psychological symptoms of dementia, poorer health outcomes and accelerated cognitive decline (Dewing and Dijk 2016; Sampson et al. 2016; Hermann et al. 2015). People with dementia in acute settings face unique risks due to cognitive and physical impairments, which complicate communication, understanding and care needs (White et al. 2016; O'Brien et al. 2020). These risks range from poor hygiene and medication mismanagement to harms, such as falls or wandering, and risks arising from mitigation strategies like restraints or involuntary care (George et al. 2013).

The fast‐paced, goal‐oriented nature of acute hospital settings can exacerbate these risks. Unlike long‐term care facilities, where care is often stable, acute hospitals prioritise rapid diagnosis, therapeutic intervention and short lengths of stay (Clissett et al. 2013). Systemic barriers, including limited resources, inadequate staff training, and a lack of interdisciplinary collaboration, significantly hinder the provision of safe and effective care (Clissett et al. 2013; Røsvik and Rokstad 2020). As a result, these systemic challenges contribute to a complex risk environment that is notably different from the community or long‐term care settings.

This scoping review examines how risk is addressed in the literature on care of people with dementia in acute settings and explores the various types of risks and risk management strategies employed in these settings. This review adopts a broad definition of risk as the probability of physical, psychological, social or emotional harm resulting from an event, condition or care practice that impacts the holistic well‐being of the individual, particularly in terms of physical safety, emotional security and social connection. The authors formulate this definition based on understanding person‐centred care and the literature on ethical care. It emphasises the multifaceted nature of risks encountered in the care of people with dementia,especially within the ethically complex and high‐pressure environments of acute hospitals.

Addressing these risks raises ethical challenges for healthcare providers who constantly have to balance patient safety with autonomy and dignity. This tension can cause moral distress among healthcare professionals (Nikunlaakso et al. 2022), adversely affecting their well‐being and work outcomes (Foà et al. 2020). This distress is not only a workforce issue but also implies a deeper structural problem in how risk is conceptualised and managed in the care of people with dementia in acute settings.

Preliminary searches by the authors suggest that the existing literature on risks in the care of people with dementia in acute settings is fragmented and often narrowly focused. There is a need for a clearer understanding of how risk is conceptualised in the care of people with dementia, and a comprehensive synthesis of the various risks and risk management strategies in acute hospital settings is needed. To address this gap, a scoping review, designed to map key concepts, types of evidence and research gaps (Peters et al. 2020; Levac et al. 2010), is more appropriate than a systematic review due to the study's broad scope and methodological diversity of the literature. This approach enables a comprehensive exploration of how risks are conceptualised, studied, and reported in the literature.

Ultimately, this review seeks to support the advancement of more holistic, person‐centred practices for the care of people with dementia in acute settings and to identify priorities for future research and policy development.

## 2 Methods

The scoping review aims to provide a broad overview of the available evidence using a systematic methodology. It was guided by the framework initially proposed by Arksey and O'Malley (2005) and further developed by the Joanna Briggs Institute (Peters et al. 2020). It includes identifying the research question, searching for relevant studies, selecting studies, charting the data, collating, summarising and reporting the results. This review adhered to the Preferred Reporting Items for Systematic Reviews and Meta‐Analyses extension for Scoping Reviews (PRISMA‐ScR) guidelines to optimise reporting (Tricco et al. 2018). The completed PRISMA‐ScR checklist is provided as File S1. The inclusion criteria and methods for the review were pre‐specified in the protocol and were registered in the Open Science Framework platform. Minor amendments were made to the protocol during the review process to reflect practical considerations. These changes were consistent with the scoping review methodology.

### 2.1 Identifying the Research Question

The research questions for this study were formulated using the Population, Concept and Context (PCC) framework proposed by the Joanna Briggs Institute (JBI) (Peters et al. 2020), as presented in Table 1.

**TABLE 1:** Population, concept and context framework.

Population	Concept	Context
People with dementia	Risks and risk management strategies associated with the care of people with dementia	Acute hospital settings

The specific research questions for the scoping review were as follows:

1. How is risk defined in the dementia literature?
2. What specific risks are identified in the dementia literature?
3. What specific risk management strategies are identified in the dementia literature?

### 2.2 Identifying the Relevant Studies

In the second stage of the scoping review, relevant studies were identified to address the research questions outlined in Stage 1. An in‐depth search of the literature was carried out using the EBSCOhost platform, covering databases Web of Science, Medline, CINAHL, Academic Search Complete and PsycINFO. The search employed the terms listed in Table 2, and the strategy was developed with expert input from specialists in dementia and ethics, alongside guidance from librarians. Medical Subject Headings (MeSH) and subject headings were used where appropriate. Boolean operators ‘OR’ and ‘AND’ were applied to combine search terms effectively.

**TABLE 2:** Search terms for database search.

Search component	Search terms
#1 Dementia	Dementia OR Alzheimer* OR ‘memory loss’ OR ‘memory deficit’: Dementia and Alzheimer's were exploded using the MeSH search strategy in Medline, CINAHL and PsycINFO to capture dementia and its subtypes
#2 Risk	Risk* OR Hazard* OR Threat* OR Safeguard* OR Protect*: Risks, hazards, and threats are included along with safeguard*/protect* to capture the risk mitigation/management strategies as well.
# 3 Hospital	Hospital* OR acute OR inpatient* OR ward* OR ‘emergency department*’ OR ‘health care’ OR healthcare
#4 Patient services	‘Patient services’ OR nurs* OR ‘clinical practice’: Nurse/nursing is also exploded as a Mesh search in CINAHL, MEDLINE and PsycInfo.

*Note:* #1 AND #2 AND #3 AND #4.

This is an example of a search from the Web of Science. Search in the Topic field (title/abstract/keywords) and limit to English.

### 2.3 Study Selection

In the third stage, studies were selected for inclusion in the scoping review. Inclusion and exclusion criteria (Table 3) were determined before the screening process.

**TABLE 3:** Inclusion and exclusion criteria.

Inclusion criteria	Exclusion criteria
Studies based on primary data (qualitative, quantitative or mixed‐method research) or systematic/scoping reviews/concept analysis that explore the following to a significant extent: Risks associated with the care of people with dementia/Identifiable subgroups or risk management/mitigation strategies in the care of people with dementia. The care of people with dementia in acute/hospital settings	Studies exclusively on disease conditions other than dementia. Studies on the care of people with dementia in nursing homes and community settings. Studies published in a language other than English Studies that are not primary or secondary research studies, including guidelines, educational materials, policy documents or other non‐peer‐reviewed content

(Covidence software n.d.) (https://covidence.org/) was used to screen and select studies. All authors were registered in Covidence and participated in all stages of the screening process. At least 50% of the articles in the Title and Abstract screening stage and 75% of the articles in the Full‐Text review stage were assessed by a minimum of two independent reviewers. Any disagreements between the two reviewers regarding article inclusion were resolved through discussion between the reviewers or, if necessary, with the involvement of an additional reviewer.

### 2.4 Charting the Data and Collating, Summarising and Reporting Results

Reviewers extracted data from the articles included in the scoping review using a data extraction tool developed by the reviewers with the aid of Covidence software. The extracted data included detailed information about the participants, study context, methods, definitions or concepts of risk, identified risk mitigation strategies (if any), types of risks, ethical issues identified or discussed (if any), and key findings relevant to the review questions. The software was used to perform dual reviewer data extraction and to review extractions for consensus.

A pilot search was conducted, and the data extraction tool was modified and revised based on the data extracted from the included studies.

AB and MC conducted the extraction, seeking support and advice from the expert co‐authors during the process. In cases of divergent extraction decisions, consensus was obtained through discussion and review meetings with the research team. The authors analysed the extracted data using an inductive approach based on content analysis, aligning with the research objectives (Elo and Kyngäs 2008).

## 3 Results

### 3.1 Search Results and Selection Process

A total of 10,337 records were imported into EndNote and then exported to Covidence for screening. After removing 6421 duplicates (6415 by Covidence and six manually), 3916 records were included for title and abstract screening. Of these, 3733 were excluded. Full‐text retrieval was attempted for 183 articles; two could not be obtained despite multiple efforts. The remaining 181 full‐text articles were assessed for eligibility, resulting in 120 exclusions. Ultimately, 61 studies were included in the review. The study selection process is illustrated in Figure 1.

**FIGURE 1:**
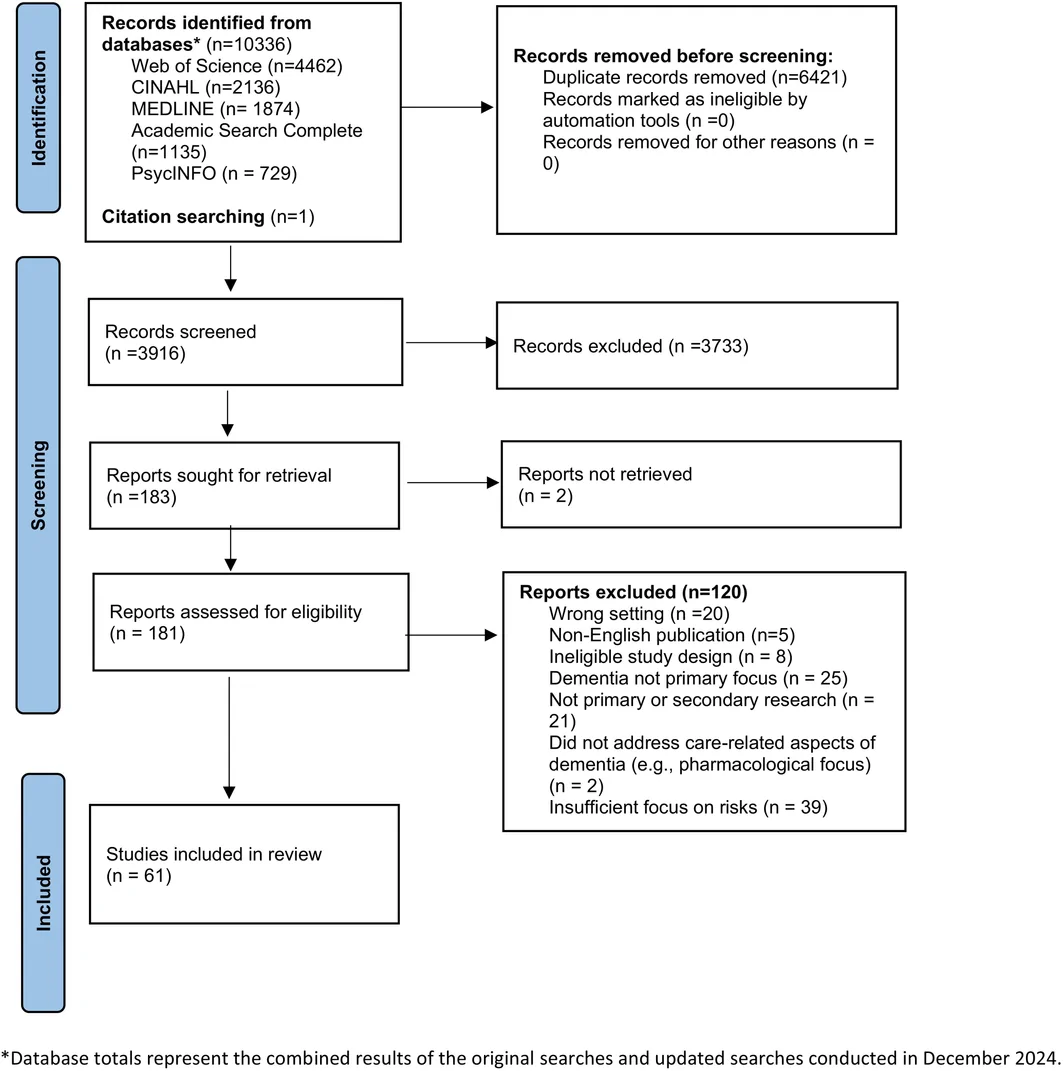
PRISMA flow diagram of the study selection process. [Colour figure can be viewed at wileyonlinelibrary.com]

The electronic database was initially searched in October 2023, and the updated search was completed in December 2024.

### 3.2 Characteristics of Included Studies

The review included 61 studies: 35 quantitative, 9 qualitative, 16 reviews and 1 mixed‐methods study, published from 1992 to 2024 (see Figure 2 below). Most studies originated from high‐income countries, with the highest representation from the USA, UK and Australia. Additional studies came from countries across Europe and Asia, including Germany, Ireland, Taiwan, Japan, Canada, China, Switzerland, the Netherlands, Austria, Norway, Finland, Spain, Denmark, Israel and Iceland. Several review papers reflected international collaborations.

**FIGURE 2:**
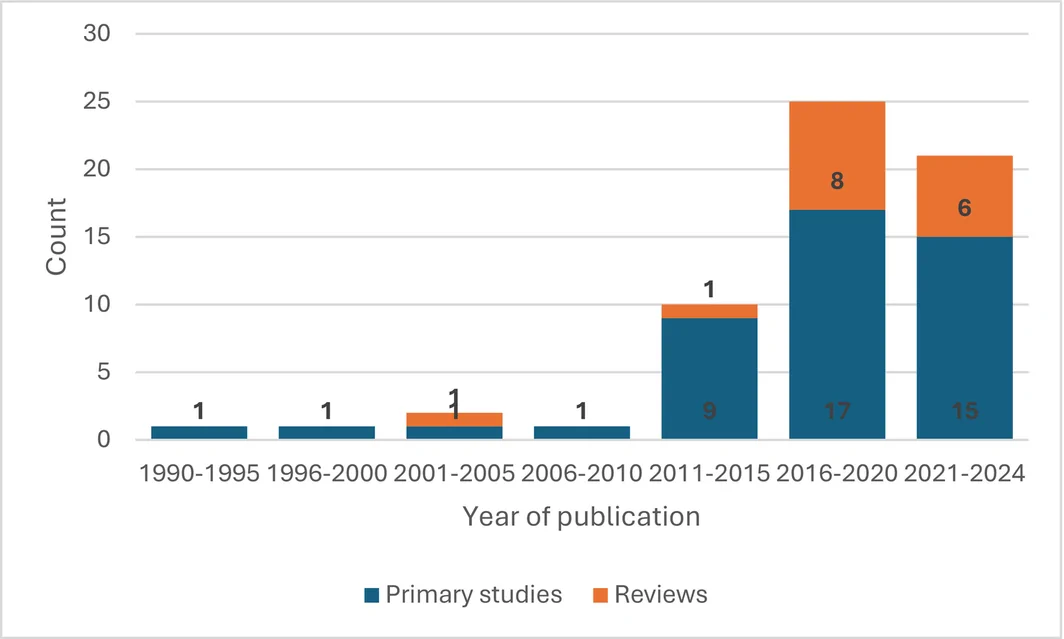
Primary studies and reviews by year of publication. [Colour figure can be viewed at wileyonlinelibrary.com]

### 3.3 How Is Risk Defined in the Dementia Literature?

*Implicit understandings of risk:* Exploring the literature on the care of people with dementia in acute settings reveals an implicit understanding of risk. Despite the critical importance of managing risk in these environments, except for one study, none of the reviewed literature provides a formal definition of risk, and most do not explore the concept in detail. Instead, risk is frequently inferred through discussions of specific issues such as patient safety, physical restraints, delayed treatment, complications and other challenges inherent in caring for people with dementia (e.g., Sullivan‐Marx 2001; DeSantis et al. 1997; Burns et al. 2020; Timmons et al. 2016; Gual et al. 2018; Makino et al. 2024; Zhang et al. 2024). Risk is thus an assumed concept, understood contextually through discussions of the negative consequences that may arise from certain clinical practices or patient vulnerabilities.

Dadich et al. (2023) define risk within the Safety‐II framework as ‘the likelihood of an unwanted event occurring.’ This conceptualisation is informed by Aven (2019) and Hollnagel et al. (2015), who emphasise the importance of addressing uncertainty and fostering resilience in complex healthcare systems. Risk is addressed as an inherent and omnipresent aspect of the care of people with dementia, necessitating continuous negotiation and management to balance autonomy and safety. This approach shifts the focus from merely preventing adverse events, as seen in traditional Safety‐I thinking, to proactively creating conditions that promote optimal outcomes and safety in complex, dynamic healthcare environments.

In some instances, the term ‘risk’ is used interchangeably with ‘safety incidents’, defined as events that compromise patient safety and result in actual or potential harm (Knuuttila et al. 2007, as cited in Kinnunen‐Luovi et al. 2014). This reflects a limited perspective on risk, emphasising only events that could adversely impact patient outcomes.

In certain studies, risk is discussed in relative terms, comparing patients with dementia to patients without dementia. For example, Bail et al. (2013) and Wu et al. (2020) highlight higher rates of mortality and morbidity following major surgery for patients with dementia. Similarly, Chróinín et al. (2024) examine hospital‐acquired complications like falls, pressure injuries, pneumonia and delirium and illustrate that patients with dementia are more vulnerable than other patients.

Some studies also conceptualise risk from the perspective of the institution or organisation caring for people with dementia. For instance, Timmons et al. (2016) and Torian et al. (1992) highlight the financial risks faced by institutions in terms of increased costs due to prolonged hospital stays and higher complications among patients with dementia.

### 3.4 What Specific Risks Are Identified in the Dementia Literature?

This scoping review identifies a wide array of risks critical to understanding and improving the care of patients with dementia in acute care settings. These risks are described in the reviewed literature and are not mutually exclusive; they are often interrelated. The specific types of risks identified, along with the risk management strategies, are outlined below. A structured overview of the types of risks, the studies that addressed them, the precipitating factors, and the proposed mitigation strategies is presented in Table S1.

#### 3.4.1 Risks of Behavioural and Psychological Symptoms of Dementia (BPSD)

The literature widely reports that BPSD, including wandering, agitation, anxiety, apathy, psychosis, delirium (Berish et al. 2024; Chróinín et al. 2024), and sleep disturbances (Brown et al. 2014), are highly prevalent among patients with dementia, significantly complicating care delivery and increasing caregiver strain. Berish et al. (2024) found that 93.9% of hospitalised patients with dementia exhibited at least one BPSD at admission, decreasing to 86.7% at discharge, with hyperactivity (76.7%), mood changes (72.3%), and psychosis (71.9%) being the most common. The persistence and severity of BPSD are associated with adverse outcomes, including increased emergency room visits, hospitalisations, and falls (Berish et al. 2024). A large proportion of people with dementia also experience disordered sleep, which significantly impacts cognition, fall risk, agitation, self‐care and overall health, often leading to institutionalisation (Brown et al. 2014).

Delirium affects up to 60% of hospitalised patients with dementia and worsens BPSD, leading to increased mortality, cognitive decline and care dependency (Voyer et al. 2006; Reynish et al. 2017; Boltz et al. 2023a; Boltz et al. 2023b). According to Boltz et al. (2023a), Boltz et al. (2023b), cognitive impairment, physical decline and antipsychotic medication partially mediate the association between delirium severity and behavioural symptoms, with a decline in physical function leading to more severe delirium and BPSD symptoms.

#### 3.4.2 Risk of Physical Restraints

The literature highlights that people with dementia are at high risk for restraint use (bedrails, belts, mittens, fixed tables, etc.) due to impaired mobility, memory deficits and cognitive declines in language, judgement and visual perception (Cotter 2005; DeSantis et al. 1997; Sullivan‐Marx 2001). Physical restraints can lead to a range of negative consequences, such as extended hospitalisations, diminished functional abilities, psychological distress, increased risk of falls, severe injuries and accelerated cognitive decline, undermining their safety goals. For patients with dementia, restraints can evoke distressing emotions, including fear, anger and humiliation, with memories of the experience persisting long after discharge. Furthermore, restraints may contribute to functional urinary incontinence by impeding patients' ability to communicate their need to use the toilet (Cotter 2005).

Despite the known adverse effects, healthcare professionals often employ restraints to manage agitation, disorientation and wandering to prevent harm to patients and others (Okumura et al. 2023; Cotter 2005). Their use is rationalised by the environmental risks, behavioural triggers such as confusion and cognitive deficits and systemic challenges, including inadequate staffing and insufficient training in the care of people with dementia (Shannon et al. 2018; DeSantis et al. 1997; Sullivan‐Marx 2001; Makino et al. 2024; Handley et al. 2017). Furthermore, organisational policies emphasising immediate safety over patient autonomy perpetuate this practice despite its known risks (Makino et al. 2024; Shannon et al. 2018).

#### 3.4.3 Risk of Inadequate Pain Management

Inadequate pain management is identified as a critical risk for patients with dementia due to challenges in accurately assessing pain in individuals with cognitive impairments (Jensen‐Dahm et al. 2016). Studies highlight the complexity of managing pain in this population, with factors contributing to underassessment and undertreatment. Jensen‐Dahm et al. (2016) found that patients with dementia undergoing surgery, particularly for hip fractures, received significantly lower opioid doses postoperatively compared to cognitively intact patients.

Fry et al. (2015) reported that older adults with cognitive impairments in emergency departments (EDs) experienced longer wait times for analgesia, often twice as long as those without cognitive impairments, exacerbating delays in pain relief. Timmons et al. (2016) revealed that nearly 25% of patients with dementia were neither asked about pain nor received formal pain assessments, increasing patient suffering and the likelihood of behavioural symptoms such as agitation and aggression.

Jonsdottir and Gunnarsson (2021) noted that nurses struggle to differentiate between pain and behavioural symptoms, compounded by knowledge deficits, workload pressure, time constraints and concerns about opioid use. Nurses often feel frustrated by physicians' lack of understanding and experience difficulties in getting them to recognise and prioritise pain in these patients, leading to delays in obtaining prescriptions and subtherapeutic treatments.

Bradford et al. (2012) emphasised that untreated pain often leads to aggression, increasing the risk of injury, care costs and compromised care quality. Recognising non‐verbal indicators of pain, such as facial expressions or behavioural changes, is crucial but frequently overlooked in patients with cognitive impairments.

#### 3.4.4 Risk of Complications and Adverse Outcomes

Hospitalised patients with dementia are significantly more vulnerable to adverse outcomes, including delirium and functional decline, due to their cognitive impairment and increased frailty (Travers et al. 2014). These adverse outcomes include both preventable and unpreventable complications. Among the preventable risks, infections are particularly prevalent. Bail et al. (2013) report high rates of urinary tract infections and pneumonia, often resulting from delayed recognition and treatment. Similarly, Chróinín et al. (2024) found that patients with dementia had a higher risk of pneumonia (OR = 1.3) compared to matched controls. Lehmann et al. (2018) identify advanced dementia as a significant predictor of adverse outcomes, including infections and mortality, which are closely associated with poor nutritional status and frailty. The findings of Torian et al. (1992) also support that patients with dementia are at high risk of treatment complications, nosocomial infections and prolonged hospital stays. Furthermore, Cheng et al. (2023) highlight that dysphagia in patients with dementia admitted to the ICU significantly increases mortality risk within 90 and 180 days, while also increasing pressure injury and aspiration pneumonia incidence.

Hospital‐acquired complications are very prevalent in patients with dementia. Geschke et al. (2019) report higher incidences of falls, fever, incontinence and pressure ulcers among patients with cognitive impairment. Chróinín et al. (2024) report an increased prevalence of falls (OR = 4.7), delirium (OR = 2.4) and pressure injuries (OR = 1.4) in this population. These patients also experience significantly prolonged hospital stays (median 9 days vs. 3 days) and higher in‐hospital mortality rates (7% vs. 5%) compared to those without dementia. Findings of Fogg et al. (2018) support this, indicating that these patients are particularly vulnerable to adverse outcomes during hospitalisation and at discharge, including in‐hospital mortality, nursing home admission, delirium, falls and infections. Reynish et al. (2017) highlight that the presence of multiple comorbidities in patients with dementia further contributes to extended hospital stays and an increased risk of complications.

The literature highlights that falls and accidents are significant risks for hospitalised patients with dementia (Kinnunen‐Luovi et al. 2014). Patient behaviours, ward routines, nursing staff shortages and environmental conditions contribute to these incidents (Kinnunen‐Luovi et al. 2014).

The literature indicates that patients with dementia undergoing surgery face a significantly higher risk of postoperative complications. Kassahun (2018) reports an increased incidence of surgical site infections (SSI), postoperative pneumonia and delirium, particularly in emergency surgeries, with in‐hospital mortality rates of 28.3% compared to 20% in patients without dementia. Similarly, Wu et al. (2020) highlight that preexisting dementia is independently associated with more significant mortality and morbidity following major surgery, with the elevated risk of mortality persisting for at least 1 year post‐surgery. These findings align with those of Diaz‐Gil et al. (2020), who report a higher prevalence of postoperative complications in patients with dementia (42.7%) compared to patients without dementia (22.8%). Common complications include SSI, urinary tract infections and respiratory issues. Additionally, these patients are at greater risk for severe medical complications such as myocardial infarction, renal failure, pneumonia, sepsis, deep wound infections, pulmonary embolism, postoperative bleeding and stroke (Diaz‐Gil et al. 2020). These risks are often exacerbated by cognitive impairments and communication challenges, hindering timely diagnosis and treatment (Diaz‐Gil et al. 2020).

Grealish, Chaboyer, et al. (2019) and Grealish, Real, et al. (2019) identify systemic failures, such as communication breakdowns between caregivers and inconsistent fall prevention protocols, as major contributors to falls. Additionally, the study highlights a lack of understanding among nurses about cognitive impairment and its influence on fall risk. Inconsistent adherence to established guidelines and insufficient family involvement also undermine the effectiveness of fall prevention strategies.

The literature identifies the risk for pressure injuries and contractures as another significant concern in hospitalised patients with dementia. Burns et al. (2020) point out that contractures are often unrecognised and untreated, leading to complications. Similarly, Jaul et al. (2019) and Bail et al. (2013) note that immobility and spasticity increase the risk of pressure ulcers. A diagnosis of dementia and prolonged hospital stays is found to significantly raise the risk of sacral ulcers (Yin et al. 2024).

The literature highlights significant risks for patients with dementia in the emergency departments (ED). Nothelle et al. (2024) report that individuals with dementia are at risk of being under‐triaged, leading to misjudgements, misdiagnoses and delayed care. Older adults with dementia typically have ED visits lasting 3 h longer than those without dementia. Han et al. (2011) found that cognitive impairment and delirium superimposed on dementia reduce the accuracy of presenting complaints and comprehension of discharge instructions. Watkins et al. (2019) emphasise that family members often feel compelled to stay at the bedside in overcrowded EDs to advocate for their loved one's needs, highlighting the significance of timely triage, private spaces and compassionate communication.

#### 3.4.5 Organisational or Healthcare Provider‐Related Risks

Inappropriate medication use, particularly antipsychotics and psychotropic drugs, poses significant risks for patients with dementia. Kennedy et al. (2022) associate antipsychotic use with prolonged hospital stays and adverse outcomes. Polypharmacy increases risks such as drug interactions and side effects (Walsh et al. 2016). Certain hypnotics, including benzodiazepines, are linked to in‐hospital fractures, while alternatives like melatonin‐receptor agonists may offer safer options (Tamiya et al. 2015). Farlow and Shamliyan (2017) warn about the limited benefits of atypical antipsychotics for managing agitation, highlighting the risks of cardiovascular issues and fractures. Similarly, Berish et al. (2024) and Dadich et al. (2023) emphasise the importance of balancing safety and patient dignity by reducing reliance on restrictive medications.

Suboptimal care practices often result from gaps in caregiver knowledge and competencies, inadequate training and unsatisfactory environments. Grealish, Chaboyer, et al. (2019) and Grealish, Real, et al. (2019) highlighted deficiencies in fall risk education, pain assessment and cognitive reorientation. Keuning‐Plantinga et al. (2022) found that nurses frequently lack the skills to manage complex behavioural symptoms of dementia despite having positive attitudes toward care. Furthermore, the lack of dementia‐specific proactive screening tools exacerbates care gaps; for instance, dysphagia is often overlooked, resulting in complications like aspiration pneumonia (Cheng et al. 2023). Nothelle et al. (2024) found that prolonged ED stays due to delayed assessments and mismanagement increase the chances of delirium and misdiagnosis.

The literature identifies several organisational and cultural issues that compromise person‐centred care for patients with dementia in acute settings. Institutional policies often prioritise acute medical tasks and safety protocols over holistic care, leading to restrictive practices. For example, using physical and chemical restraints to manage behaviours such as aggression or wandering emphasises immediate safety rather than long‐term well‐being (Moyle et al. 2011; Digby et al. 2018). These risk‐averse approaches contribute to ethical concerns, eroding patient dignity and autonomy (Digby et al. 2018).

Chróinín et al. (2024) emphasise that hospitalised patients with dementia face increased risk of adverse outcomes due to modifiable factors, including the hospital environment and staff‐related factors (e.g., nursing ratios, skill mix and burnout). In emergency care, systemic inefficiencies heighten risks for patients with dementia.

Cultural inefficiencies, such as the ‘pad culture’ described by Northcott et al. (2022), illustrate how institutional pressures prioritise efficiency over patient‐centred continence care. The routine use of continence pads, regardless of individual needs, has been linked to increased distress and complications, such as pressure ulcers. The study emphasises that involving patients in decisions about toileting can enhance outcomes and maintain dignity. Similarly, Digby et al. (2018) report that in acute care, fundamental aspects of care, such as personal hygiene and patient engagement, are often deprioritised in favour of clinical tasks and technological interventions.

#### 3.4.6 Nutritional Risks

Malnutrition is a significant issue among people with dementia who are hospitalised, with over 72% malnourished and 25% at risk for further deterioration (Lin et al. 2023). Patient‐specific challenges, such as dementia‐related eating difficulties and behavioural symptoms, as well as systemic barriers, including caregiver stress, time constraints, inadequate training and unsuitable dining environments, aggravate this risk (Liu et al. 2020).

Dysphagia is a common concern among elderly patients with dementia, particularly in hospital settings, including intensive care units. Dysphagia significantly increases the risk of adverse health outcomes, including mortality, aspiration pneumonia and pressure injuries (Cheng et al. 2023).

#### 3.4.7 Risk to Personhood and Dignity in Acute Care

The literature highlights that institutional cultures in acute settings often prioritise efficiency and safety over person‐centred care, leading to risks to dignity and autonomy (Digby et al. 2018). Task‐oriented care models frequently reduce individuals to passive recipients of treatment, overlooking their emotional and social well‐being. The ‘pad culture’ in continence management, where incontinence pads are used as a convenience rather than based on individual needs, demonstrates how institutional priorities may undermine dignity (Northcott et al. 2022).

The literature further identifies that restrictive safety measures, such as physical and chemical restraints, are commonly used to manage behavioural symptoms and prevent wandering. However, these interventions are associated with adverse outcomes, including distress, decreased mobility and loss of autonomy (Shannon et al. 2018). Zhang et al. (2024) describe absolute dignity as the intrinsic worth of individuals, whereas caregiving environments affect relative dignity by limiting autonomy and self‐expression.

### 3.5 What Specific Risk Management Strategies Are Identified in the Dementia Literature?

This section synthesises the various risk management strategies employed in acute hospital settings as identified in the reviewed literature. The following main themes have emerged.

#### 3.5.1 Targeted Screening and Tailored Interventions

The literature highlights the significance of early detection of risks to prevent complications and adverse outcomes in patients with dementia. Studies suggest that targeted screening tools and comprehensive geriatric assessments can effectively identify risks such as delirium, BPSD, polypharmacy, malnutrition (Cheng et al. 2023), pain (Fry et al. 2015) and wandering (Sheth et al. 2014). Early intervention through family‐centred care for delirium has been identified as a valuable approach to reduce its severity and duration. Standardised tools, including the Mini‐Cog, Wandering Screening Tool, Braden Scale, bedside swallowing assessments and validated fall risk tools, can help in identifying cognitive impairments, dysphagia, pressure ulcer risks and fall risks, especially in emergency and intensive care settings (Lehmann et al. 2018; Nothelle et al. 2024; Geschke et al. 2019; Cheng et al. 2023; Yin et al. 2024). The prevention and management of agitation and aggression are also critical in acute care to protect patient safety and reduce iatrogenic risks (Akrour et al. 2022).

Tailored preoperative and postoperative care for patients with dementia to improve surgical outcomes is also highlighted as crucial. Kassahun (2018) recommends conservative strategies in non‐emergency surgical cases to avoid unnecessary interventions, whereas Wu et al. (2020) and Cheng et al. (2023) emphasise the importance of comprehensive risk stratification to identify high‐risk patients and provide tailored postoperative care for optimal recovery.

#### 3.5.2 Dementia‐Friendly Environmental Modifications

Dementia‐friendly environments contribute to reduced adverse outcomes, increased autonomy, improved patient safety and comfort and enhanced caregiver satisfaction (Chróinín et al. 2024; Lehmann et al. 2018; Dadich et al. 2023; Zhang et al. 2024).

Dementia‐friendly ward designs, characterised by calming colours, adequate lighting and designated spaces for activities and rest, have been shown to mitigate agitation, sleep disturbances and delirium (Makino et al. 2024; Akrour et al. 2022; Brown et al. 2014).

Modifications, including low beds and enhanced lighting, are also reported to reduce fall risks, thus decreasing the perceived necessity for physical restraints (Cotter 2005). Safe ward layouts incorporating wayfinding cues and dementia‐friendly design features create safer environments that accommodate movement while minimising confusion and fall risks (Handley et al. 2017). Dementia‐friendly environments facilitate the adoption of strategies such as ‘watchful wandering’ to enable safe, supervised mobility, thereby preserving dignity while minimising restrictive measures (Shannon et al. 2018).

The creation of dementia‐friendly dining spaces supports patients in improving their nutritional intake. A low stimulation setting, including aromatic food, natural light and structured social interactions, reduces mealtime stress. Furthermore, a supportive socio‐cultural environment, encouraging engaging conversations and consistent tablemates, optimises mealtime experiences for individuals with dementia (Liu et al. 2020).

#### 3.5.3 Non‐Pharmacological Interventions

Non‐pharmacological interventions like music therapy and Simulated Presence Therapy (SPT) were found to significantly reduce agitation and reliance on antipsychotics (Cummings et al. 2023; Duan et al. 2024). SPT, which utilises personalised stimuli, showed improvements in both agitation and cognitive function, although further validation is needed due to limited sample sizes.

For pain management, non‐pharmacological interventions like physical therapy and relaxation techniques were emphasised. These interventions provided effective pain relief while minimising the reliance on opioids, which carry additional risks for patients with dementia (Timmons et al. 2016).

Medication reviews are also vital in minimising polypharmacy and identifying safer alternatives to antipsychotics and benzodiazepines, helping to minimise adverse outcomes and ensure safer medication management for patients with dementia (Farlow and Shamliyan 2017; Tamiya et al. 2015).

#### 3.5.4 Person‐Centred, Family‐Inclusive Care

Some studies emphasise the importance of person‐centred approaches for individuals with dementia. Implementing these models in acute settings can reduce adverse outcomes related to BPSD and enhance caregiver satisfaction (Chróinín et al. 2024). Person‐centred, restraint‐free care promotes patient autonomy and dignity, prioritising non‐restrictive strategies over physical restraints and sedation (Makino et al. 2024; Cotter 2005). Engaging in structured physical activity can enhance functional recovery and mitigate behavioural symptoms in patients with dementia by preserving muscle strength and fostering independence (Boltz et al. 2023a).

Person‐centred care frameworks are integral in addressing nutrition‐related risks. Targeted mealtime assistance for individuals improves nutritional intake and reduces the risk of deterioration (Lin et al. 2023). Tailoring meals to patient preferences, offering familiar foods and maintaining structured mealtime routines enhance food consumption and minimise agitation (Liu et al. 2020).

Person‐centred continence care, which involves individuals in decision‐making regarding their bowel and bladder function, supports independence and overall well‐being (Northcott et al. 2022). This approach illustrates the broader principle of balancing patient safety with respect for personhood.

Family involvement is a key component of person‐centred care (Karrer et al. 2021). Interventions such as Family‐Centred Function‐Focused Care (Fam‐FFC) promote collaboration between nurses, patients and families, leading to improved recovery and continuity of care (Boltz et al. 2023a; Boltz et al. 2023b). Studies also highlight the significance of involving families in decision‐making and risk identification to reduce falls and restraint use (Grealish, Chaboyer, et al. 2019; Grealish, Real, et al. 2019; Stockwell‐Smith et al. 2020; Moyle et al. 2011). Liu et al. (2020) emphasise the importance of caregiver training and support during mealtimes to address malnutrition risks, while Liu et al. (2020) identify caregiver stressors such as time pressure and lack of training as barriers to optimal nutritional care. The role of family members as active contributors to patient safety is also emphasised by Dadich et al. (2023).

#### 3.5.5 Enhancing Workforce Competency and Interdisciplinary Collaboration

The literature highlights the critical role of skilled, collaborative healthcare teams in managing dementia‐related risks. Gerontological nurses play a key role in detecting and managing delirium and other complications through comprehensive assessments (Schnitker et al. 2020). Interdisciplinary collaboration facilitates coordinated care, particularly for pain and behavioural symptoms, while empowering nurses as advocates to promote holistic, patient‐centred care (Jonsdottir and Gunnarsson 2021; Okumura et al. 2022).

Building staff competence in the care of people with dementia is vital (Berish et al. 2024; Chróinín et al. 2024; Lehmann et al. 2018; Karrer et al. 2021). Dementia‐specific training enhances nurses' abilities to manage challenging behaviours, recognise delirium early and reduce reliance on restrictive practices (Keuning‐Plantinga et al. 2022; Chen et al. 2024). Programs that emphasise empathy, effective communication and de‐escalation techniques improve the management of behavioural symptoms without restraint use (Makino et al. 2024; Handley et al. 2017). Training to identify nonverbal pain cues and differentiate them from other behavioural expressions strengthens clinical decision‐making (Jonsdottir and Gunnarsson 2021). Caregiver education on dementia‐friendly nutritional support strategies improves nutritional outcomes (Liu et al. 2020). Additionally, training ED nurses to prioritise older adults with dementia while triaging and incorporating family input into assessments can support timely and appropriate care (Watkins et al. 2019).

#### 3.5.6 Organisational Support and Systemic Reforms

Institutional support and systemic changes are identified as significant for sustainable improvement in the care of people with dementia in acute settings. These include implementing least‐restraint policies, mobility‐promoting practices and protocols aligned with person‐centred principles (Abraham et al. 2022; Makino et al. 2024).

Leadership engagement and adequate resource allocation are critical for reinforcing person‐centred care and ensuring continuity of staff training. Strong organisational support enhances the implementation of best practices (Handley et al. 2017; Berish et al. 2024).

Additionally, creating socio‐cultural environments that encourage patient autonomy, especially during mealtimes, can improve nutrition intake and overall well‐being (Liu et al. 2020).

#### 3.5.7 Multicomponent Interventions

The literature emphasises the importance of employing diverse risk management strategies, considering the complex and multifaceted nature of dementia‐related risks. Strategies that combine staff training, environmental modifications, interdisciplinary teamwork and individualised patient interventions effectively manage BPSD, reduce falls and prevent delirium (Cotter 2005; Stockwell‐Smith et al. 2020). Stockwell‐Smith et al. (2020) identify effective fall prevention strategies encompassing family engagement, comprehensive risk assessments, technology‐assisted nursing observation, regular medication reviews and non‐pharmacological interventions for managing delirium. Preventing restraint use in the care of people with dementia requires addressing physiological, psychosocial, environmental and systemic factors, alongside institutional challenges (Cotter 2005).

## 4 Discussion

The literature on the care of people with dementia in acute settings addresses risk in a fragmented manner, focusing on specific risks and mitigation strategies. To the best of our knowledge, this is the first review to systematically consolidate various risks and risk management strategies in the care of people with dementia in acute settings, providing a comprehensive reference for nurses and healthcare professionals. It offers valuable insights into how risk is defined, managed and mitigated in the care of people with dementia within acute hospital settings.

One key finding of this review is the absence of a clear, standardised definition of risk within the literature. Risk is understood implicitly in the context of individual studies—whether related to patient safety, institutional practices or environmental factors (DeSantis et al. 1997; Sheth et al. 2014). This lack of clarity limits the comparability of findings and challenges how healthcare professionals interpret and manage risk. The ambiguity in risk conceptualisation also makes it challenging to stratify and prioritise risks effectively in acute settings. Establishing a theoretical and conceptual foundation for risk in the care of people with dementia could help in a better understanding of risk in this population and optimise care practices.

The review also highlights a predominant focus on physical risks such as falls, restraints and malnutrition, while psychological, social and spiritual risks are comparatively underexplored. Issues like social isolation, loss of identity and spiritual distress are often neglected, despite having a considerable impact on the well‐being of individuals with dementia (Northcott et al. 2022). These findings align with those of Stevenson et al. (2016), who also noted a tendency to prioritise physical risks over other dimensions of risk. This narrow approach contradicts Kitwood (1997) Dementia Care Model and suggests challenges in translating person‐centred care principles into practice within acute settings. The prevailing task‐oriented, safety‐driven model prioritises physical harm prevention over emotional security, social connection and dignity (Brooker 2007; Digby et al. 2018). Psychological risks such as anxiety and depression receive limited attention, with compliance‐based interventions replacing non‐pharmacological, person‐centred strategies (Shannon et al. 2018). Paradoxically, safety interventions like physical restraints, which are intended to protect patients, may inadvertently cause psychological distress, autonomy loss and threats to dignity (Cotter 2005). This reactive approach to risk management ultimately overlooks long‐term consequences for patients' emotional and psychological well‐being.

Another recurring theme identified in the literature is the perception of individuals with dementia as risky or disruptive in hospital settings, which leads to a focus on controlling disruptions to routine rather than person‐centred care. Instead of recognising dementia‐related behaviours as expressions of unmet needs, these behaviours are often considered risks to manage, leading to further distress and disengagement (Handley et al. 2017). This risk‐averse approach, neglecting the root cause of these issues, reinforces stereotypes of individuals with dementia as passive rather than active participants in their care.

A holistic approach that balances immediate safety with long‐term well‐being is essential to tackle these challenges. Expanding risk assessments to include psychological, social, and spiritual factors will promote person‐centred care and improve the safety and quality of life of patients with dementia.

A key element of this shift is the concept of positive risk‐taking, also known as risk enablement, which aims to balance the potential benefits of taking risks with the drawbacks of excessive restrictions. Overemphasising risk avoidance can impose unnecessary limitations, whereas a more balanced approach can enhance patient confidence, self‐satisfaction and independence (Manthorpe and Moriarty 2011; Morgan and Williamson 2014). Rather than viewing risk solely as a threat to be minimised, positive risk‐taking reframes it as an opportunity for empowerment and identity preservation. Despite its potential advantages, this concept remains under‐represented in the reviewed literature.

However, some studies highlight its significance in upholding patient dignity and improving care quality. For instance, Moyle et al. (2011) underscore the importance of holistic risk management that prioritises the individual's autonomy and dignity while addressing safety concerns. Research by Handley et al. (2017) and Makino et al. (2024) also demonstrates that reducing reliance on restrictive measures can improve patient outcomes and minimise harm. The Safety‐II framework advocated by Dadich et al. (2023) emphasises the importance of creating positive conditions that allow dignified choices. Similarly, Yamaguchi et al. (2019) and Cummings et al. (2023) emphasise balancing risk prevention with respecting patient preferences, enabling individuals to maintain autonomy while addressing potential hazards.

Positive risk‐taking can enhance emotional well‐being by enabling engagement in meaningful activities while managing hazards. Zhang et al. (2024) highlights the benefits of empowering individuals with dementia to express their preferences in day‐to‐day care activities and engage in meaningful activities, fostering dignity and independence. These strategies align with person‐centred care principles, fostering dignity and individuality while reducing reliance on restrictive measures (Northcott et al. 2022; Shannon et al. 2018).

However, systemic barriers, such as staffing shortages, risk‐averse institutional cultures and limited dementia‐specific training, reinforce restrictive care models, limiting opportunities for autonomy‐focused interventions (Makino et al. 2024; Handley et al. 2017; Manthorpe and Moriarty 2011). Addressing these challenges requires a fundamental shift in organisational priorities, including investments in workforce development, ongoing training, and a broader cultural transformation within healthcare institutions. Without substantial funding, organisational support and strategic prioritisation, efforts to embed positive risk‐taking and person‐centred approaches are unlikely to succeed.

The review also finds that the role of caregivers in supporting patients with dementia in acute hospital settings is not fully acknowledged in the reviewed literature. Although some studies acknowledge their contributions to reducing falls, improving nutrition, enhancing communication and upholding dignity (Grealish, Chaboyer, et al. 2019; Grealish, Real, et al. 2019; Liu et al. 2020; Moyle et al. 2011; Zhang et al. 2024; Watkins et al. 2019), most studies do not sufficiently explore their role within acute care settings.

Caregivers often play a vital role in interpreting patient needs, facilitating communication and contributing to decision‐making, particularly when patients experience cognitive or communicative impairments. Caregiver involvement has been shown to improve psychosocial well‐being and reduce agitation, anxiety and depression in people with dementia (Merrilees 2016; Rababa et al. 2023).

Despite the significant role of caregivers in providing optimal care for patients with dementia, their role is not well acknowledged in the literature. This reflects the situation in clinical settings where the perspectives of caregivers are rarely incorporated into hospital‐based risk assessment frameworks, despite their relevance to patient safety and care quality.

Moreover, caregivers themselves face emotional fatigue, physical strain and psychological burden, which may compromise their ability to provide adequate support (Etters et al. 2008; Brites et al. 2025). Structured interventions such as the REACH II programme demonstrate that caregiver well‐being can be enhanced through education, health tracking and personalised guidance (Elliott et al. 2010), highlighting the need to consider caregiver support as part of patient safety strategies.

The literature thus suggests that caregiver involvement remains an underused resource in the care of patients with dementia in acute hospital settings. Future research should examine models that formally incorporate caregiver perspectives into risk assessment and care planning, while also addressing the support needs of caregivers. This dual focus may contribute to safer, more person‐centred care and improve outcomes for both patients and families.

This review also finds that, although the literature extensively explores various risks and management strategies, it largely neglects the ethical implications of these practices, particularly regarding autonomy, dignity and well‐being. (DeSantis et al. 1997; Sheth et al. 2014; Gual et al. 2018). Only a few studies provide insight into this aspect. Ethical issues, such as the tension between autonomy and safety, are common in the care of people with dementia. Some harm‐prevention strategies may paradoxically cause distress in patients. For example, though designed to enhance safety, identifiable vests for patients at risk of wandering can stigmatise and dehumanise individuals, affecting their social identity (Chen et al. 2024). While physical restraints are meant to prevent injury, they often result in fear, humiliation and cognitive decline, contradicting person‐centred care principles (Shannon et al. 2018; Okumura et al. 2023).

These examples underscore the ethical tensions inherent in safety‐driven interventions, which directly affect autonomy, a principle central to person‐centred care. Respect for autonomy is explicitly acknowledged in some studies (Zhang et al. 2024; Yamaguchi et al. 2019; Digby et al. 2018), often framed as an ethical obligation to respect the preferences and personhood of people with dementia. However, these references depict autonomy as a static or binary concept, either present or absent, without considering how it may evolve over the course of dementia. In earlier stages, individuals may retain decision‐making capacity, and supporting their choices helps preserve identity and agency. As cognitive decline progresses, however, traditional models of autonomy may become less applicable. In such cases, supported decision‐making, where individuals are assisted in expressing their preferences, offers a more appropriate and ethical framework (Daly et al. 2018).

Relational autonomy builds on this by recognising the influence of caregivers, family members, and cultural context in shaping care decisions (Novy et al. 2023). Respecting both current and past wishes, life history and cultural values allows care to reflect the relationships and identities that shape the individual. Several studies acknowledge the role of families in the care of patients with dementia (Digby et al. 2018; Boltz et al. 2023a; Boltz et al. 2023b; Watkins et al. 2019), and while they do not explicitly frame this involvement within a relational autonomy model, they do indicate that family input can influence care decisions and outcomes. Notably, some studies (e.g., Boltz et al. 2023a; Boltz et al. 2023b) also highlight the exclusion of families from decision‐making processes, which may limit advocacy for patient autonomy and obscure the relational dimensions of ethical care.

These findings suggest that current hospital‐based care of patients with dementia may lack the suitable conceptual tools and adequate ethical guidance necessary to navigate the complexities of autonomy in practice. Integrating models that incorporate supported decision‐making, precedent autonomy, and attention to the person's present well‐being (Daly et al. 2018; Soofi 2022) could offer more flexible and compassionate approaches, particularly in advanced stages of dementia. Embedding such frameworks into acute care settings may help balance safety with dignity and move beyond procedural understandings of autonomy toward more inclusive, person‐centred care.

Additionally, antipsychotic medications, commonly prescribed for behavioural symptoms, pose significant ethical concerns due to their links to increased mortality and cognitive decline Farlow and Shamliyan (2017).

Ethical issues also arise in pain management, where delays and inadequate treatment exacerbate suffering, worsen behavioural symptoms, and reduce the quality of life (Jensen‐Dahm et al. 2016; Fry et al. 2015). Similarly, antipsychotic medications used for behavioural symptom management pose serious ethical concerns, as they are linked to increased mortality and cognitive decline Farlow and Shamliyan (2017). Ethical prescribing of these medications requires transparent communication, informed consent and collaborative decision‐making with patients to ensure that potential benefits outweigh the risks (Beauchamp and Childress 2019).

Additionally, resource constraints within healthcare systems contribute to implicit care rationing, affecting patients and caregivers. Staff shortages, inadequate dementia‐specific training, and the undervaluation of nursing roles further compromise care quality and increase caregiver burnout (Bail et al. 2013). Addressing these systemic issues requires ethical frameworks prioritising equitable resource distribution, workforce development and organisational support.

Ethical concerns are particularly pressing in end‐of‐life care, where conflicts arise between prolonging life and alleviating suffering. Nurses frequently advocate for compassionate pain relief and dignified end‐of‐life care, yet organisational policies and family expectations may complicate decision‐making (Jonsdottir and Gunnarsson 2021; Brorson et al. 2014). These issues highlight the need for patient‐centred, ethically sound end‐of‐life practices.

To address these gaps in ethical engagement, future research should strengthen the understanding of the ethical issues associated with risks and risk management in acute hospital settings. Exploring healthcare professionals' perspectives and identifying the barriers they face in delivering ethically sound, person‐centred care for people with dementia is essential. This review also highlights the need for empirical research to explore, in greater depth, the risks associated with the care of these patients in acute hospital settings.

Integrating ethical considerations into the policies related to the care of people with dementia, ensuring that policy development, staff training and clinical decision‐making incorporate ethical guidelines, is important for the provision of person‐centred ethical care for patients with dementia. Organisational or management support is vital in ensuring these are met. By embedding ethical principles into the care policies, research, training and practice, healthcare institutions can uphold autonomy, dignity and justice, fostering a more humane, equitable and person‐centred model of care for people with dementia.

### 4.1 Strengths and Limitations

This review provides a comprehensive and systematic synthesis of risk and risk management strategies in the care of people with dementia within acute hospital settings. To the best of our knowledge, this is the first review to systematically map the spectrum of risks and mitigation strategies in the care of people with dementia in acute settings, highlighting the complex challenges across these environments. By integrating both primary and secondary research evidence, this review offers a nuanced understanding of how risks are defined, managed and mitigated. It also identifies key gaps in existing literature, particularly regarding ethical engagement and the under‐representation of positive risk‐taking. This review lays a valuable foundation for future research and policy development, serving as a critical resource for policymakers and hospital administrators seeking to improve the care for people with dementia in acute settings.

While this review offers valuable insights, it has certain limitations. Notably, the exclusion of non‐English language publications introduces a potential language bias, which may limit the cultural and contextual diversity of the findings. Although this decision was pragmatic given resource constraints, it restricts access to potentially valuable insights from non‐English‐speaking regions, including diverse intercultural experiences and alternative care practices for people with dementia globally. Future reviews should consider multilingual inclusion strategies to address this gap.

Secondly, the omission of grey literature and unpublished studies may have limited the comprehensiveness of this review, potentially excluding emerging practices, policy reports and innovations in the care for people with dementia that are not captured in peer‐reviewed sources.

Although 3916 articles were screened and 61 studies were included, the decision to exclude grey literature was based on resource constraints, as this study was not funded and was primarily conducted by a PhD student. While the included studies vary in methodological rigour, this scoping review's primary focus was to identify risks in the care of people with dementia rather than perform an in‐depth quality analysis. To bolster the credibility of the findings, two independent reviewers assessed at least 50% of the articles during the screening process; however, the resources available limited comprehensive review procedures.

## 5 Conclusion

This scoping review highlights significant conceptual gaps and limitations in the understanding and management of risks associated with the care of people with dementia in acute settings. A predominant focus on physical safety risks often overlooks the psychological, social and spiritual aspects of well‐being. It leads to a culture prioritising control over autonomy and individualised support, resulting in a task‐driven approach to care. When academic literature still represents the care of people with dementia predominantly in terms of efficiency and control rather than autonomy and personhood, achieving meaningful change in clinical practice becomes challenging.

Institutional factors and safety policies contribute to restrictive care models that misinterpret the behaviours of patients with dementia as threats, leading to distressing interventions. The review underscores the urgent need for systemic changes and policy reforms, advocating for a greater emphasis on positive risk‐taking and risk enablement to enhance independence and emotional well‐being.

In addition to clinical risk management, this review highlights significant ethical concerns in the care of people with dementia, particularly regarding the balance between safety, dignity, autonomy and equitable access to care. Despite the increased complexity of decision‐making in acute hospital settings, ethical discussions in dementia literature remain limited. It is essential to explore the ethical perspectives of healthcare professionals, especially nurses, who navigate complex decisions while balancing autonomy, dignity, and safety in the care of people with dementia. This exploration is crucial in the care of people with dementia, where there is a heightened risk of patients losing their sense of personhood and dignity due to their increased vulnerability.

A balanced approach to risk that prioritises person‐centred care, long‐term well‐being, and ethical considerations alongside safety concerns can foster a more supportive and humane model of care. This approach is not only beneficial but is critical to upholding the dignity, autonomy, and overall well‐being of patients with dementia admitted to acute hospital settings.

## Author Contributions

A.B., K.I., E.C. and B.G. conceptualised and designed the study. A.B. conducted the literature search. A.B. and M.C. screened the studies and extracted data with contributions from K.I., E.C. and B.G. K.I., E.C. and B.G. guided the study methodology and execution. A.B. drafted the manuscript. K.I., E.C. and B.G. critically reviewed and provided intellectual input on the manuscript. All authors read and approved the final manuscript.

## Funding

The authors have nothing to report.

## Ethics Statement

The authors have nothing to report.

## Conflicts of Interest

The authors declare no conflicts of interest.

## Supporting information


**File S1:** Preferred reporting items for systematic reviews and meta‐analyses extension for scoping reviews (PRISMA‐ScR) checklist.

**Table S1:** Synthesis of risks and risk management strategies in the care of people with dementia in acute hospital settings.

## Data Availability

The dataset supporting this scoping review (extracted data from included studies) is stored in the Open Science Framework (OSF) repository and is available in read‐only mode via the following OSF link https://osf.io/r7esm/?view_only=e753c4b2b1e24c53ab34d066e4500441. Researchers interested in accessing the full dataset may contact the corresponding author, Ashitha Bhaskaran (ashitha.eb@gmail.com). Requests will be reviewed on a case‐by‐case basis and may require a data sharing agreement.
