# Supplementary material for: Understanding Risks in the Care of People With Dementia in Acute Hospital Settings: Definitions, Types and Management Strategies—A Scoping Review
Source: J Clin Nurs. 2026 Aug 8;35(11):4708–24. doi: 10.1111/jocn.70433 (PMC13629285; doi:10.1111/jocn.70433)
Supplement: Supplementary file 2 [file JOCN-35-4708-s002.docx]

**Supplementary Table 1
Synthesis of Risks and Risk Management Strategies in the Care of People with Dementia in Acute Hospital Settings**

| **Type of Risk** | **Associated Risks Addressed** | **Studies Addressing These Risks** | **Details of Risk/Harm** | **Related Adverse Outcomes** | **Precipitating Factors** | **Risk Mitigation Strategies** |
| --- | --- | --- | --- | --- | --- | --- |
| Behavioural and Psychological Symptoms of Dementia (BPSD) | Wandering, agitation, anxiety, apathy, sleep disturbances, psychosis, and delirium | Berish et al. (2024); Chróinín et al. (2024); Boltz et al. (2023); Akrour et al. (2022); Brown et al. (2014); Cummings et al. (2023); Sheth et al. (2014); Duan et al. (2024); Voyer et al. (2006); Reynish et al. (2017); Schnitker et al. (2020); Travers et al. (2014) | High prevalence of BPSD among hospitalised patients; delirium is also common and may exacerbate symptoms | Increased emergency visits, hospitalisations, falls, mortality, cognitive decline, and caregiver burden | Multifactorial: biological, psychosocial, social, and environmental; hospital environments exacerbate BPSD | Early screening tools (e.g., wandering screening tool); dementia-friendly environments that minimise environmental overstimulation; nonpharmacological interventions (e.g., music therapy, simulated presence therapy); person-centred care models |
| Physical Restraints | Agitation, disorientation, wandering, falls | Abraham et al. (2022); Cotter (2005); Shannon et al. (2018); Makino et al. (2024); Handley et al. (2017); DeSantis et al. (1997); Okumura et al. (2023); Sullivan-Marx (2001) | High prevalence among patients with impaired mobility and behavioural symptoms | Functional decline, psychological distress (e.g., fear, humiliation), cognitive deterioration, incontinence | Organisational focus on immediate safety, staffing and training deficiencies | Staff education in non-restrictive strategies, empathy, and de-escalation; environmental modifications; interdisciplinary collaboration; standardised restraint-free care protocols |
| Inadequate Pain Management | Underassessment and undertreatment of pain, delayed analgesia | Jensen-Dahm et al. (2016); Fry et al. (2015); Timmons et al. (2016); Bradford et al. (2012); Jonsdottir et al. (2021) | Patients with dementia often receive significantly less pain relief; inadequate evaluation of pain in ED settings | Agitation, aggression, injuries, complications, misdiagnosis, increased care costs | The high-pressure environment of EDs, difficulty interpreting nonverbal signs of pain by staff, knowledge gaps, and interprofessional communication issues | Use of dementia-specific pain assessment tools; staff training; interdisciplinary collaboration; non-pharmacological approaches (e.g., physical therapy, relaxation strategies) |
| Complications and Adverse Outcomes | Infections (e.g., UTI, pneumonia), pressure injuries, delirium, post-op complications, dysphagia, mortality | Bail et al. (2013); Chróinín et al. (2024); Lehmann et al. (2018); Kassahun (2018); Wu et al. (2020); Burns et al. (2020); Diaz-Gil et al. (2020); Fogg et al. (2018); Grealish et al. (2019a, 2019b); Gual et al. (2018); Jaul et al. (2019); Kinnunen-Luovi et al. (2014); Travers et al. (2014); Schnitker et al. (2020); Stockwell-Smith et al. (2020); Tamiya et al. (2015); Boltz et al. (2023); Nothelle et al. (2024); Yin et al. (2024); Cheng et al. (2023) | Elevated risk of hospital-acquired complications and increased morbidity | Prolonged hospital stays, increased mortality | Under-triage of patients with dementia in EDs, cognitive/communication challenges, behavioural symptoms, dementia-related biological challenges, system-level issues (e.g., routines, staff shortages, staff competency issues) | Fostering dementia-sensitive/friendly environments in emergency and acute care settings; effective screening and early triage of patients with dementia in emergency settings; targeted screening for early identification of cognitive impairments and complications using dementia-specific tools; staff training; person- and family-centred care; technology-assisted observation; regular medication reviews; and fall and delirium prevention strategies |
| Organisational or Healthcare Provider-Related Risks | Inappropriate medication use, suboptimal care practices, and institutional cultural issues | Kennedy et al. (2022); Walsh et al. (2016); Tamiya et al. (2015); Farlow et al. (2017); Dadich et al. (2023); Berish et al. (2024); Grealish et al. (2019); Ko-Xin Chen et al. (2024); Zhang et al. (2024); Cheng et al. (2023); Lehmann et al. (2018); Keuning-Plantinga et al. (2022); Moyle et al. (2011); Northcott et al. (2022); Han et al. (2011); Watkins et al. (2019) | Adverse outcomes from polypharmacy, inappropriate prescribing, cognitive and functional decline, and task-oriented risk-averse care practices that undermine person-centred dementia care | Increased falls, deterioration of care quality, risk of fractures and cardiovascular issues related to antipsychotics, compromised patient autonomy and dignity | Risk-averse culture, efficiency-focused protocols, skill mix issues, staff burnout, lack of organisational support and resources for effective dementia care delivery. | Regular medication reviews; safer pharmacologic alternatives (e.g., melatonin receptor agonists); institutional leadership support; staff training; shared decision-making; and dementia-friendly environments |
| Nutritional Risks in Dementia Care | Malnutrition, dysphagia | Lin et al. (2023); Liu et al. (2020); Cheng et al. (2023) | A high proportion of hospitalised patients with dementia are reported to be malnourished | Weight loss, frailty, dehydration, aspiration pneumonia, pressure injuries, mortality | Eating difficulties resulting from cognitive impairment, behavioural issues, systemic issues (e.g., time constraints of staff, caregiver stress and limited knowledge/skills, dining environment) | Person-centred mealtime support; caregiver training and support; appropriate diet texture modification; patient-informed decisions; and dementia-friendly dining spaces (low-stimulation setting, including aromatic food, natural light, and structured social interactions) |
| Risk to Personhood and Dignity | Loss of autonomy and dignity due to restrictive, task-oriented care approaches | Digby et al. (2018); Northcott et al. (2022); Shannon et al. (2018); Zhang et al. (2024); Watkins et al. (2019) | Institutional efficiency-driven practices undermine person-centred care, e.g., routine use of incontinence pads regardless of individual need | Psychological distress, increased agitation, loss of patient self-identity and autonomy | Efficiency-focused culture, overuse of restraints, staffing limitations, lack of dementia-specific training and support | Individualised, person-centred continence care; restraint alternatives; dementia-friendly environments; mobility programs that support positive risk-taking; staff training and organisational support |

*Note. This table summarises the risks encountered by people with dementia in acute hospital settings and the associated risk management strategies.*

*BPSD = Behavioural and Psychological Symptoms of Dementia; ED = Emergency Department. Studies are cited to demonstrate supporting evidence.*
